# Maximizing Electric Power through Spectral‐Splitting Photovoltaic‐Thermoelectric Hybrid System Integrated with Radiative Cooling

**DOI:** 10.1002/advs.202206575

**Published:** 2023-02-07

**Authors:** Jiangfeng Guo, Xiulan Huai

**Affiliations:** ^1^ Institute of Engineering Thermophysics Chinese Academy of Sciences Beijing 100190 P. R. China; ^2^ Department of Chemical Engineering Imperial College London London SW7 2AZ UK; ^3^ Nanjing Institute of Future Energy System Nanjing 211135 P.R. China; ^4^ School of Engineering Science University of Chinese Academy of Sciences Beijing 100049 P. R. China

**Keywords:** concentrated spectral splitting, multi‐junction cells, photovoltaic, radiative cooling, solar energy, thermoelectric generator

## Abstract

As zero‐emission technologies, a daytime radiative cooling (RC) strategy developed recently, and photovoltaic (PV) and thermoelectric (TE) technologies have aroused great interest to reduce fossil fuel consumption and carbon emissions. How to integrate these state‐of‐the‐art technologies to maximise clean electricity from the sun and space remains a huge challenge, and the limit efficiency is still unclear. In this study, a spectral‐splitting PV‐TE hybrid system integrated with RC is proposed to maximise clean electricity from the sun and space without any emissions. For the sun acting as a typical constant heat‐flux heat source, the current thermoelectric theory overestimates the thermoelectric efficiency highly since the theory is based on constant temperature‐difference conditions. A new theory based on heat‐flux conditions is employed to achieve maximum thermoelectric efficiency. The PV‐TE hybrid system with RC is superior to the conventional hybrid system, not only in terms of higher efficiency but also in its 24‐h operation capacity. In a system with a single‐junction cell, the total efficiency with 30 suns (39.4%) is higher than the theoretical PV efficiency at 500 suns (38.2%). In a hybrid system with four‐junction cells, total efficiency is over 65% which is superior to most current photoelectric and thermal power systems.

## Graphical abstract

A concentrated spectral‐splitting photovoltaic‐thermoelectric hybrid system integrated with radiative cooling is proposed to maximize clean electricity from the sun and space without any emissions. The system with a low concentration ratio (*C*_r_) can achieve conventional solar cells’ efficiency with a high *C*_r_, and achieve 24‐h operation with maximum efficiency exceeding 65%, which outperforms most current photoelectric and thermal power systems.

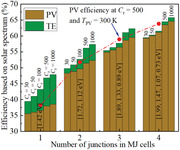

## 1 Introduction

To tackle the challenges relating to environmental pollution and climate change, clean and renewable energy is increasing its penetration in the energy landscape, aiming at reducing the use of conventional fossil fuels.^[^ ^1^ ^]^ Among all renewables, solar energy is the most important one thanks to its simple accessibility, cleanliness, and unlimited potential. Solar energy can be directly converted into electricity through photovoltaic (PV) cells without noise or moving components.^[^ ^2^ ^]^ The efficiency limit of a conventional single‐junction solar cell is around 33%, also known as Shockley–Queisser (S‐Q) limit,^[^ ^3^ ^]^ which is much lower than Landsberg (93%) and Carnot limits (95%),^[^ ^4^ ^]^ since a large portion of the solar spectrum cannot be used in the photon‐electricity conversion.

The unused portion of the solar spectrum dissipates as waste heat in PV cells, including the photon energy below/above the PV cell's bandgap energy (*E*_g_). It is acknowledged that the PV efficiency decreases relatively by 4.5% and the ageing rate of PV cells doubles for an increase of 10 °C in the PV cell's temperature (*T*_PV_).^[^ ^5^ ^]^ Therefore, enhancing spectrum utilization and reducing waste heat generation to fill the gap between S‐Q and Carnot limits have attracted significant attention in recent decades, and various design strategies and concepts such as spectral‐splitting (SS) technology and multi‐junction (MJ) solar cells have been proposed.^[^ ^6^ ^]^ The photons with energy below *E*_g_ can be split via SS and further exploited through other options to enhance the full‐spectrum utilization. MJ solar cells, which consist of multiple stacked PV cells with different *E*_g_, can absorb the appropriate band that matches the *E*_g_ of a specific cell, thereby enhancing the overall efficiency.^[^ ^7^ ^]^

Another way to improve the solar utilization efficiency of PV cells is to utilize the waste heat through a thermoelectric generator (TEG) to produce additional electricity. In a hybrid photovoltaic‐thermoelectric (PV‐TE) system, it is generally to direct the ultraviolet (UV) and visible bands of the solar spectrum to the PV cell, while the infrared (IR) band is directed to a heat absorber that acts as the hot side of TEG via concentrated SS.^[^ ^8^ ^]^ Elsarrag et al.^[^ ^9^ ^]^ conducted theoretical and experimental studies on a hybrid PV‐TE system to address the feasibility of using such systems in large‐scale applications. Yin et al.^[^ ^10^ ^]^ proposed an optimization method to maximize solar energy harvest for SS PV‐TE systems, with results showing that there existed an optimal temperature distribution in the TE subsystem. The optimal cutoff wavelength was the maximum available wavelength of GaAs PV cell and was slightly affected by the convective heat transfer coefficient of the cooling system when the thermoelectric figure of merit was small, and the optimal cutoff wavelength decreased as the figure of merit increased. Alnajideen and Min^[^ ^11^ ^]^ experimentally investigated a hybrid PV‐TE system with SS strategy, and the results showed that the overall efficiency was increased by 6.3% due to the harvesting of IR spectrum using TEG, and a further increase in efficiency could be achieved using light concentration. Another commonly used method is to place TE modules directly underneath PV panels, with PV panels acting as the hot sides of TE modules. Khan et al.^[^ ^12^ ^]^ performed an experimental study of such configurations, and the results showed that the *T*_PV_ could be decreased by 3 K, and consequently, the total output power was increased by 19% and the overall solar‐to‐electric efficiency was increased by 17% with respect to the bare PV panels. The TE modules that operated alone under solar irradiation conditions were also studied. Peak efficiencies of 4.6% and 7.4% could be achieved with solar irradiances of 1 and 211 kW m^−2^, respectively, as reported by Kraemer et al.^[^ ^13^ ^]^

Regardless of the structural form, a heat sink as the cold side is required in TEG, and various types are available. The common cooling methods include air cooling,^[^ ^14^ ^]^ liquid cooling (e.g., water),^[^ ^15^ ^]^ phase change materials,^[^ ^16^ ^]^ etc., and the ambient is usually employed as the final cold source, resulting in a higher temperature in the cold side of TEG than the ambient temperature. As a clean and sustainable cooling technology that has been developed recently, daytime radiative cooling (RC) can emit heat to outer space with a temperature of 3 K through the “atmospheric window” (wavelength *λ* ranges from 8 to 13 µm) without any additional energy consumption, so as to achieve a lower cooling temperature than the ambient temperature.^[^ ^17^ ^]^ Raman et al.^[^ ^18^ ^]^ devised a photonic radiative cooler that could reflect >90% of the incident sunlight and emit heat through the atmospheric window, with experimental results showing that a temperature drop of 4.9 °C could be achieved relative to the ambient temperature at midday and demonstrating the feasibility of using RC as a clean cooling approach. Chen et al.^[^ ^19^ ^]^ reported an average temperature drop of 37 °C relative to the ambient temperature was experimentally achieved after a 24‐h day–night cycle, with a maximum temperature drop of 42 °C at midday.

PV panel has sufficient area to face the sky and is very suitable for the use of RC technology. Zhu et al.^[^ ^20^ ^]^ integrated RC with a bare crystalline Si PV panel, demonstrating that the PV panel's temperature could be reduced by 18.3 °C at 1 sun. Also, based on a crystalline Si cell, a PV system integrated with SS and RC was proposed by Gao et al.,^[^ ^21^ ^]^ and the experimental results indicated that the panel's temperature could be reduced by 23.2 °C when the wind speed was 3 m s^−1^ and by 68.1 °C when there was no wind. Wang et al.^[^ ^22^ ^]^ conducted multiple simulations and experiments to investigate the impact of radiative cooling on solar cells, they found RC performance varied significantly with changes in wind speed, heat load, and design, the temperature drop of 36 °C was observed in a sealed chamber relative to a system without RC. However, Li et al.^[^ ^23^ ^]^ experimentally and theoretically investigated the effect of RC on a commercial PV module, with results indicating that the temperature reduction and efficiency improvement were very slight, since the encapsulating layers (like glass cover) had high thermal emissivity. This issue could be partially addressed by optimized coating, Li et al.^[^ ^24^ ^]^ reported that the temperature of an encapsulated PV module could be reduced by 5.7 °C, using a multilayer structure with strong reflectivity in UV and 1.3–1.8 µm bands and high emissivity in the 4–25 µm band. Besides, the actual effect of RC is affected by relative humidity, cloud cover, wind speed, latitude, etc.,^[^ ^25^ ^]^ which remains to be further studied before its large‐scale application. Fan et al.^[^ ^26^ ^]^ presented a TEG‐RC system, and the results showed that the power density of TEG could be increased by 53% relative to the scenario with regular blackbody emitters.

The theory of TEG operating at constant temperature‐difference conditions has been established and has been widely adopted to estimate the TEG electrical power and efficiency.^[^ ^27^ ^]^ The theory assumes that TEG works at a constant‐temperature heat source, and its operating temperature difference does not change with the operating modes. However, the actual temperature difference varies with the operating modes (open circuit, closed circuit, short circuit, etc.), resulting in much lower efficiency than the theoretical efficiency. In addition, as a typical constant heat‐flux condition, the temperature of the solar absorber that acts as the hot side of TEG varies with the properties of TEG. Therefore, how to accurately predict the maximum TE efficiency is crucial to exploit the potential of integrating TEG with PV panels in practical applications. Previous research mainly focuses on the applications of RC in PV panels or TE modules alone, high efficiency is hard to achieve, especially for stand‐alone TE modules. Hybrid PV‐TE systems achieve higher efficiency than stand‐alone PV panels, while the capability of generating electricity during both daytime and nighttime without energy storage remains a challenge. Integrating RC with a hybrid PV‐TE system at concentrated sunlight conditions, and the limit efficiency and operation mode remain unclear. In this study, we develop a methodology and a model to combine the state‐of‐the‐art SS methodology, RC, and PV‐TE hybrid technologies to maximize electricity generation from the sun and space without any emissions. The impacts of RC, concentration ratio, and material properties of PV panels on the maximum efficiency of the hybrid system are analyzed. This study provides insight into the SS, PV‐TE, and RC technologies as well as useful guidance for the design and optimization of relevant components and systems.

## 2 Results

### 2.1 Single‐Junction PV‐TE Hybrid System

**Figure 1:**
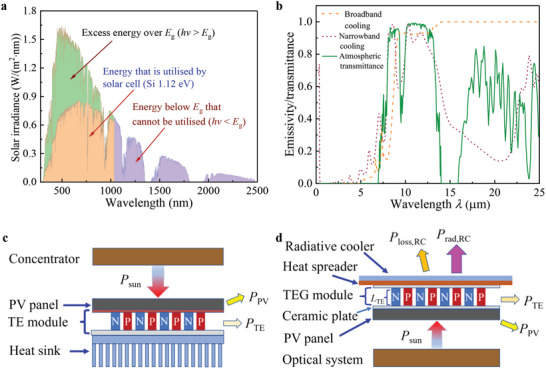
Solar spectrum, the emissivity of radiative cooler, PV‐TE hybrid systems. a) The AM1.5 solar spectrum and the part of the spectrum irradiance that can be utilized by Si solar cell (*E*_g_  =  1.12 eV). b) Emissivities of narrowband cooling in ref. [28] and broadband cooling in ref. [20] and atmospheric transmittance in ref. [31]. c) A hybrid PV‐TE system with a heat sink. d) A hybrid PV‐TE system with radiative cooling.

Photons with energy below *E*_g_ cannot be electrically utilized by PV cells and are thus converted to thermal energy. Similarly, for photons with energy over *E*_g_, the excess energy that cannot be used by PV cells is also ultimately converted to thermal energy (i.e., the thermalization loss), as shown in **Figure** **1**a. In both cases, this leads to the increasing operating temperature of PV cell (*T*_PV_), and decreasing PV efficiency (*η*_PV_) and lifespan. In a radiative cooler, the emissivity plays a crucial role, which can be classified as either narrowband cooling or broadband cooling as shown in Figure 1b. In a narrowband cooling, the high emissivity should coincide with the “atmospheric window” as much as possible, while the emissivity in other bands (*λ* < 8 µm or *λ* > 13 µm) should be as low as possible to achieve a temperature lower than the ambient temperature (*T*_a_). In broadband cooling, the emissivity should be zero in the solar spectrum and as high as possible in other bands as shown in Figure 1b (the emissivity in broadband cooling comes from ref. [20]), achieving a large cooling power with a temperature higher than *T*_a_. A temperature lower than *T*_a_ is desired to benefit TEG, especially at night, so narrowband cooling is adopted in this study. The optimized multilayer structure of narrowband cooling adopted here was proposed in ref. [28], whose materials include MgF_2_, TiO_2_, SiN, SiO_2_, etc. The optical properties of these materials can be found in literature, and the emissivity of the multilayer structure could be obtained through the characteristics matrix method.^[^ ^29^ ^]^ Multilayer films of these materials are commonly used for radiative cooling, and their effectiveness has been verified by experiments.^[^ ^18^, ^30^ ^]^

In a conventional PV‐TE hybrid system as shown in Figure 1c, a PV panel is placed on the top acting as the hot side of TEG, and a heat sink is placed at the bottom acting as the cold side of TEG. TE module consisting of numerous P‐N thermocouples is placed in the middle, to generate electricity using the temperature difference between the solar panel and heat sink. Heat is dissipated from the heat sink to the environment via convection and radiation, making the temperature of the heat sink higher than *T*_a_. In this study, radiative cooling is used to replace the heat sink as the cold side of TEG to create a lower cold‐side temperature than *T*_a_. Radiative cooling needs to face the sky, so it is placed on the top as shown in Figure 1d. A solar panel is placed at the bottom as the hot side of TEG, while the TEG is placed in the middle to generate electricity using the temperature difference between the PV panel and RC. Compared with the system in Figure 1c, the light path in the system in Figure 1d is slightly more complicated, and it needs a reflector to direct sunlight to the solar panel from bottom to top. A similar light path scheme has been experimentally verified to have good performance in ref. [22].

The sunlight is AM1.5 spectral irradiation shown in Figure 1a, and the PV efficiency is calculated based on the method of detailed balance limit first introduced by Shockley and Queisser.^[^ ^3^ ^]^ The *T*_PV_ is not fixed as in the classical analysis, but the temperature at which the PV cells reach heat balance with the surrounding components and environment (see Equation (17) in the Experimental Section) in this study. The PV efficiency can be written as

(1) $$\eta_{\mathrm{PV}}=\frac{P_{\mathrm{PV}}}{\int_{0}^{\infty }C_{r}A_{\mathrm{PV}}I_{\mathrm{AM}1.5}d\lambda }$$

where *P*_PV_ denotes PV power, *I*_AM1.5_ refers to the AM1.5 standard spectral irradiance,^[^ ^32^ ^]^ *A*_PV_ is the PV panel's area, and *C*_r_ is the concentration ratio which is defined as

(2) $$C_{r}=\frac{A_{\mathrm{op}}}{A_{\mathrm{PV}}}$$

where *A*_op_ is the aperture area of the optical concentrator. Analogous to the concentration ratio, the cooling ratio (*R*_c_) is defined as the ratio of the equivalent area of the cooler (including the heat sink) to the area of the PV panel

(3) $$R_{c}=\frac{A_{\mathrm{RC}}}{A_{\mathrm{PV}}}$$

where *A*_RC_ denotes the area of radiative cooler, and also denotes the area of the heat sink in Figure 1c for the convenience of comparison. When the temperature of radiative cooler (*T*_RC_) is higher than *T*_a_, heat is dissipated to outer space and the surrounding environment via radiation and convection. When *T*_RC_ is lower than *T*_a_, heat is dissipated to outer space via radiation, while the surrounding environment will act as a heat source. Detailed information about RC can be found in Section *Radiative Cooling* in the Experimental Section. TEG needs to connect an external load in practical applications, and the ratio of external load resistance to internal resistance can be written as

(4) $$s=\frac{R_{L}}{R_{i}}$$

where *R*_L_ is the external load resistance and *R*_i_ denotes the internal resistance of TEG. This ratio *s* has an important impact on the performance of TEG in the constant heat‐flux theory adopted in this study, which is to overcome the invalidation of the current constant temperature‐difference theory, and the details will be discussed in the following sections. One structural parameter that has an important influence on the TE power is the ratio of cross‐sectional area to the length in the TE module

(5) $$AL=\frac{N_{\mathrm{TE}}A_{\mathrm{TE}}}{L_{\mathrm{TE}}}$$

where *N*_TE_ is the number of P‐N thermocouples, *A*_TE_ is the cross‐sectional area of a thermocouple, and *L*_TE_ is the length of P‐N thermocouples. For the convenience of comparison, the equivalent efficiency of TEG in a hybrid PV‐TE system is defined as

(6) $$\eta_{TE\_\mathrm{eq}}=\frac{P_{\mathrm{TE}}}{\int_{0}^{\infty }C_{r}A_{\mathrm{PV}}I_{\mathrm{AM}1.5}d\lambda }$$

where *P*_TE_ is the TE power. Therefore, the total efficiency of the hybrid PV‐TE‐RC system can be written as

(7) $$\eta_{\mathrm{PV}+TE}=\frac{P_{\mathrm{PV}}+P_{\mathrm{TE}}}{\int_{0}^{\infty }C_{r}A_{\mathrm{PV}}I_{\mathrm{AM}1.5}d\lambda }$$

(8) $$\eta_{\mathrm{tot}}=\frac{P_{\mathrm{PV}}+P_{\mathrm{TE}}+P_{\mathrm{TE}\_n}}{\int_{0}^{\infty }C_{r}A_{\mathrm{PV}}I_{\mathrm{AM}1.5}d\lambda }$$

where *P*_TE_n_ denotes the TE power at night.

**Figure 2:**
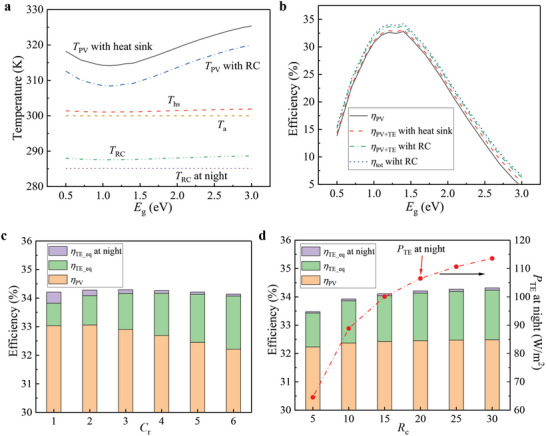
Temperature and efficiency in the hybrid systems in Figure 1. a) The temperature in the two PV‐TE systems in Figure 1 as a function of bandgap energy at conditions of *C*_r_  =  1 and *R*_c_  =  20. b) The efficiency in the two PV‐TE systems in Figure 1 as a function of *E*_g_, at conditions of *C*_r_  =  1 and *R*_c_  =  20. c) Efficiencies in the PV‐TE‐RC system as a function of *C*_r_ at *R*_c_ = 20. d) Efficiencies in the PV‐TE‐RC system and TE power at night as a function of *R*_c_ at *C*_r_ = 5. Other conditions: *AL*  =  0.014 m and *s* = 2.

The temperature and efficiency as a function of bandgap energy in the two systems in Figure 1c,d, under the same conditions of *T*_a_ = 300 K, *s* = 2, *E*_g_  =  1.4 eV, *AL*  =  0.014 m, *h*_c_ = 5 W m^−2^ K^−1^ (convective heat transfer coefficient, representing the heat exchanged with the atmosphere via convection, can be reduced by windshield or vacuum significantly), are presented in **Figure** **2**. The *T*_PV_ is 5.6 K lower in the PV‐TE‐RC system than in the PV‐TE system in Figure 1c on average in the range of *E*_g_, indicating the RC has better cooling effectiveness than the heat sink and the PV panel performs better in the PV‐TE‐RC system. The temperature of heat sink (*T*_hs_) in the PV‐TE system in Figure 1c is 1.4 K (on average) higher than *T*_a_ over the range of *E*_g_, while the *T*_RC_ in the system in Figure 1d is 12 K (on average) lower than *T*_a_ during daytime. Of note is that *T*_RC_ is 285.1 K which is nearly 15 K lower than *T*_a_ at night. The total efficiency of PV panel and TEG (*η*_PV+TE_) in the conventional system in Figure 1c (the maximal value is 33.1%) is increased by 4.4% relatively (on average) compared to *η*_PV_ over the range of *E*_g_, while *η*_PV+TE_ in the PV‐TE‐RC system (maximal value is 33.8%) is increased by 9.3% relatively compared to *η*_PV_ on average. More importantly, TEG can also generate electricity at night in the PV‐TE‐RC system since *T*_RC_ is lower than *T*_a_. Considering the electricity generation of PV panel, TEG at both daytime and nighttime, the total equivalent efficiency (*η*_tot_) of the PV‐TE‐RC hybrid system is increased by 11.8% relatively compared to *η*_PV_ on average, and the maximum value reaches 34.2%, which is higher than the S‐Q limit (≈33%) and the *η*_PV+TE_ in the conventional PV‐TE system (33.1%) under 1 sun.

In the PV‐TE‐RC system in Figure 1d, *C*_r_ and *R*_c_ represent the intensity of “hot source” and “cold source,” and their influences on the efficiencies and TE power at night are demonstrated in Figure 2c,d. At *R*_c_  =  20, with an increment of *C*_r_, *η*_PV_ increases to a peak (33%) at *C*_r_ = 2 and then decreases due to the increasing *T*_PV_, while *η*_TE_eq_ monotonously increases from 0.8% at *C*_r_ = 1 to 1.9% at *C*_r_ = 6. *C*_r_ has no impact on TE power at night, but the *η*_TE_eq_ decreases from 0.4% at *C*_r_ = 1 to 0.06% at *C*_r_ = 6. Although increasing *C*_r_ is beneficial to TEG, *η*_PV_ dominates in the total efficiency (*η*_tot_), and *η*_tot_ reaches a peak (34.3%) at *C*_r_ = 3 and then decreases quickly. Because the TE module placed above the PV panel is not conducive to heat dissipation, when the deterioration effect of rising *T*_PV_ on PV panel outweighs the benefit to TEG, *η*_tot_ will inevitably decline. At *C*_r_  =  5, with the increment of *R*_c_ from 5 to 30, *η*_TE_eq_ increases from 1.2% to 1.8%, *η*_TE_eq_ at night rises from 0.05% (65 W m^−2^) to 0.08% (114 W m^−2^), and *η*_PV_ rises from 32.2% to 32.5%, leading to the increase in *η*_tot_ from 33.5% to 34.3%. Clearly, the increasing *R*_c_ is very conducive to the PV‐TE‐RC system, especially to TEG, while the increasing *C*_r_ is not always good for the efficiency of the whole system.

### 2.2 Spectral‐Splitting PV‐TE System

**Figure 3:**
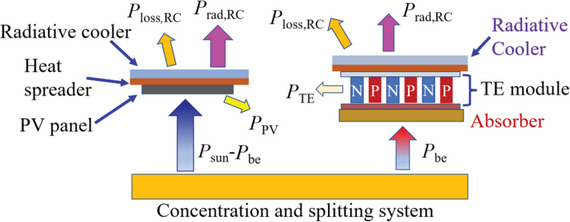
A concentrated spectral‐splitting (SS) single‐junction PV‐TE‐RC system. Photons with energy above *E*_g_ are directed to PV cell with matching *E*_g_, the excess thermalization heat is dissipated through a radiative cooler placed above the PV panel. The separated spectrum is directed to a TE module that utilizes a radiative cooler to obtain a lower temperature than *T*_a_.

With the increase of *C*_r_, *T*_PV_ increases remarkably, leading to the decreasing *η*_tot_ in the PV‐TE‐RC system in Figure 1d. Under high *C*_r_ conditions, it is necessary to separate the spectrum with photon energy lower than *E*_g_ for reduction of *T*_PV_ as well as full‐spectrum utilization as shown in **Figure** **3**. The methodology to harvest solar energy by splitting solar spectrum and directing each band to the matching convertor was proposed first by Jackson.^[^ ^33^ ^]^ So far, various mechanisms and methods have been proposed for spectral‐splitting of sunlight, such as refractive and diffractive methods, etc., and the details about these methods can be found in some excellent review papers.^[^ ^34^ ^]^ The photons with energy above *E*_g_ are directed to PV panel with matching *E*_g_, and the thermalization heat in the PV panel is dissipated through a radiative cooler placed above the PV panel, to avoid *T*_PV_ being too high under high *C*_r_, and *T*_PV_ is restricted to be less than 373 K in this study. The separated photons with energy below *E*_g_ are directed to the absorber, whose temperature (*T*_ab_) can be very high (>400 °C) under high *C*_r_, there are many ways to use the high‐temperature absorber for power generation, such as TEG, thermal power generation, etc. Among them, TEG is one of the most convenient and environmentally friendly power generation methods with the lowest maintenance cost, so TEG is selected here. To further improve the efficiency of TEG, a radiative cooler is used as the cold side to obtain a lower temperature than *T*_a_ as shown in Figure 3.

According to the current thermoelectric theory under constant temperature‐difference conditions, the maximum conversion efficiency can be obtained by^[^ ^35^ ^]^

(9) $$\eta_{\mathrm{TE},\max}=\left(1-\frac{T_{c}}{T_{h}}\right)\frac{\sqrt{1+ZT}-1}{\sqrt{1+ZT}+T_{c}/T_{h}}$$

with

(10) $$s_{\mathrm{opt}}=\sqrt{1+ZT}$$

where *T*_h_ and *T*_c_ denote the temperatures on the hot and cold sides of TEG, *Z* is figure‐of‐merit, and *ZT* denotes dimensionless figure‐of‐merit, which can be written as

(11) $$ZT=\frac{\alpha^{2}}{\rho \lambda }T$$

where *α* denotes Seebeck coefficient, *ρ* is electrical resistivity, *λ* denotes thermal conductivity, and *T* is the absolute temperature of the TE module that is often estimated to be (*T*_h_+*T*_c_)/2.

**Figure 4:**
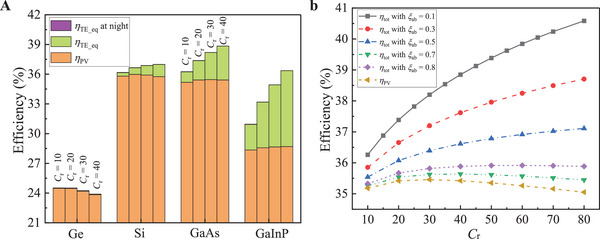
Efficiencies in the SS PV‐TE‐RC system as a function of *C*_r_ with various PV materials. a) Efficiencies as a function of *C*_r_ for different materials (Ge, Si, GaAs, and GaInP) at *ξ*_ab_ = 0.1. b) Efficiencies as a function of *C*_r_ with different heat loss coefficients of absorber at *E*_g_ = 1.42 eV. Other conditions: *R*_c_ =  25 and *s*  =  2.

Of note is that Equation (9) is assumed that temperature difference (∆*T*) across TEG is constant (i.e., constant‐temperature heat source, *T*_h_), and the ratio *s* has no effect on ∆*T*. However, *s* has an important influence on the temperature difference across TEG, and the heat source (solar absorber) in the system in Figure 3 can be regarded as a constant heat‐flux heat source rather than a constant‐temperature heat source, making Equation (9) invalid. To address the issue in the current TE theory, a newly developed theory based on constant heat‐flux conditions is adopted here,^[^ ^36^ ^]^ the details about this new theory are presented in Section *Thermoelectric Efficiency under Constant Heat‐Flux Conditions* in the Experimental Section. Based on the commonly used PV materials Ge (*E*_g_  =  0.66 eV), Si (*E*_g_  =  1.12 eV), GaAs (*E*_g_  =  1.42 eV), and GaInP (*E*_g_  =  1.81 eV), the efficiencies in the system in Figure 3 as a function of *C*_r_ are presented in **Figure** **4**, at conditions of *R*_c_ = 25, *ξ*_ab_ = 0.1 (heat loss coefficient of absorber), and *s* = 2. As *C*_r_ increases from 10 to 40, *η*_PV_ decreases from 24.5% to 23.8% for Ge, reaches a peak of 36% at *C*_r_ = 20 for Si, reaches a peak of 35.5% at *C*_r_  =  30 for GaAs, and increases from 28.4% to 28.7% for GaInP. Figure 2c indicates that *η*_PV_ increases and then decreases as *C*_r_ increases, thus the optimal *C*_r_ at which *η*_PV_ reaches maximum increases as the material's *E*_g_ increases in Figure 4a. *η*_TE_eq_ increases as *C*_r_ rises, and grows greatly as the material's *E*_g_ increases, since the separated spectrum with photon energy below *E*_g_ increases as the material's *E*_g_ increases. For example, *η*_TE_eq_ is 0.04% for Ge on average over the range of *C*_r_ from 10 to 40, while is 5% for GaInP on average which increases more than 100‐fold_r_. This indicates that the selection of suitable PV material is crucial to this SS PV‐TE‐RC system. Although the Si PV panel has the highest *η*_PV_ among the four materials, the system including GaAs PV panel has the highest *η*_tot_ which is 37.7% on average over the range of *C*_r_ from 10 to 40. The absolute difference of *η*_tot_ between the systems including Si PV panel and GaAs PV panel increases as *C*_r_ increases, from 0.1% at *C*_r_ = 10 to 1.9% at *C*_r_ = 40. The *η*_TE_eq_ at night becomes very small under high *C*_r_, whose mean value is 0.01% over the range of *C*_r_ from 10 to 40. The *η*_TE_eq_ is very small in the system including Ge PV panel, *η*_tot_ is almost the same with *η*_PV_ and decreases as *C*_r_ grows due to increasing *T*_PV_. However, *η*_tot_ increases as *C*_r_ grows for the other three PV materials, since the proportion of TE power increases.

To further analyze the tendency of *η*_tot_, the effect of the heat loss coefficient of absorber (*ξ*_ab_) on *η*_tot_ is presented in Figure 4b, at *R*_c_ = 25 and *E*_g_ = 1.42 eV. *η*_PV_ increases to a peak of 35.5% at *C*_r_ = 30, and then decreases. *η*_tot_ also increases and then decreases in the cases of *ξ*_ab_ = 0.8 and 0.7, while the *C*_r_ corresponding to the maximal *η*_tot_ becomes higher as *ξ*_ab_ becomes lower, which is 40 at *ξ*_ab_ = 0.8 and 60 at *ξ*_ab_ = 0.7. The decreasing *ξ*_ab_ means the increasing heat flux across TEG, leading to increasing *η*_TE_eq_. Therefore, *η*_tot_ increases monotonously without a peak when *ξ*_ab_ is 0.5 or lower in the range of *C*_r_ from 10 to 80. In the range of *C*_r_ from 10 to 80, a 10% relative increment of *η*_tot_ is achieved (on average) compared to *η*_PV_ at *ξ*_ab_ = 0.1, even when half of the absorbed heat is lost (i.e., *ξ*_ab_ = 0.5), *η*_tot_ is still increased by 3.6% relatively on average. Various measures can be taken to reduce heat loss, such as placing the absorber in a vacuum environment to reduce convective heat loss, and increasing absorptivity in the solar spectrum and reducing emissivity in the emitted spectrum through suitable coating, etc. There are no moving parts or working mediums in the PV‐TE‐RC system, so it is easy to place this system in a closed or vacuum environment to reduce *h*_c_ and *ξ*_ab_.

### 2.3 Thermoelectric Conversion Efficiency in Solar Energy

**Figure 5:**
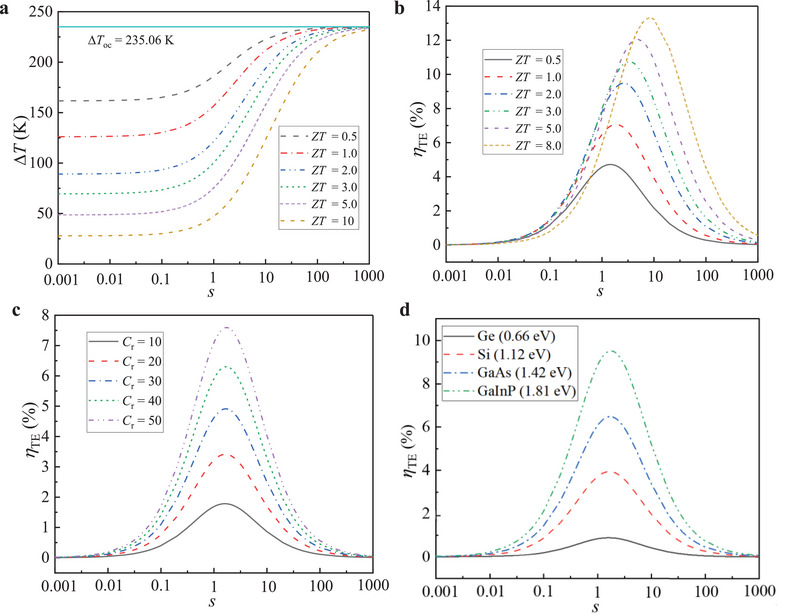
Temperature difference and TE efficiency as a function of *s* under various conditions in constant heat‐flux theory. a) Temperature difference of TEG in a closed circuit as a function of *s* with various *ZT*, at *C*_r_  =  40 and *E*_g_ = 1.4 eV. b) TE efficiency as a function of *s* with various *ZT*, at *C*_r_ = 40 and *E*_g_ = 1.4 eV. c) TE efficiency as a function of *s* with various *C*_r_, at *Z* = 2 × 10^−3^ K^−1^ and *E*_g_ = 1.4 eV. d) TE efficiency as a function of *s* with various PV materials, at *C*_r_ = 40 and *Z*  =  2×10^−3^ K^−1^. Other conditions: *T*_c,oc_ = 285.4 K and *ξ*_ab_ = 0.1.

The theoretical efficiency of PV cells has been widely studied, while the TE theoretical efficiency under solar irradiance conditions is still misunderstood, which is one of the keys in the PV‐TE‐RC system. The presence of Peltier heat leads to a lower temperature difference (∆*T*) across TEG in a closed circuit than in an open circuit, so the practical efficiency will be highly overestimated using the constant ∆*T* theory (i.e., Equation (9)).^[^ ^36^ ^]^ In the constant heat‐flux theory, the ∆*T* in a closed circuit (∆*T*_cc_) and the *η*_TE_ as a function of *s* at conditions of *C*_r_ = 40, *T*_c,oc_ = 285.4 K (the temperature on the cold side in an open circuit, is generally assumed unchanged in theoretical analysis), *ξ*_ab_ = 0.1, and *E*_g_ = 1.4 eV, are presented in **Figure** **5**. In an open circuit, only Fourier heat exists across TEG and the ∆*T* across TEG (∆*T*_oc_) remains unchanged. Clearly, the Peltier heat and Joule heat strengthen as *ZT* increases in a closed circuit, and the ∆*T* across TEG decreases. For a given *ZT*, the ∆*T* across TEG increases as the ratio *s* increases. In the case of *s* = 0, TEG is in a short circuit, the current is the maximum, and the ∆*T* across TEG is the smallest, and also decreases as *ZT* increases due to the stronger Peltier heat and Joule heat. In the case of *s*  =  ∞, TEG is in an open circuit, the ∆*T* across TEG is ∆*T*_oc_.

In constant heat‐flux theory, the *η*_TE_ increases first as the ratio *s* increases, reaches a peak and then decreases as shown in Figure 5b. The peak value of *η*_TE_ increases as *ZT* increases, from 4.7% at *ZT* = 0.5 to 13.3% at *ZT* = 10. The optimal ratio *s*_opt_ that corresponds to the peak of *η*_TE_ for a given *ZT*, also increases as *ZT* rises, from 1.5 at *ZT* = 0.5 to 8 at *ZT* = 10. At *ZT* = 1.0, the *s*_opt_ is 1.9, that is different from the constant ∆*T* theory in which the maximal output power corresponds to *s*_opt_  =  1 and the maximal *η*_TE_ corresponds to Equation (10). Figure 5a,b shows that decreasing *s* leads to reducing ∆*T* across TEG, resulting in the reduction of voltage difference across TEG, while the decreasing *s* results in an increase of current. Therefore, there exists an *s*_opt_ in which current and voltage reach trade‐off, leading to a peak of *η*_TE_. At *E*_g_ = 1.4 eV and *Z*  =  2 × 10^−3^ K^−1^, the peak value of *η*_TE_ increases significantly as *C*_r_ increases as shown in Figure 5c, from 1.8% at *C*_r_ = 10 to 7.6% at *C*_r_ = 50. While the *s*_opt_ corresponding to the maximum *η*_TE_ slightly increases as *C*_r_ increases with constant heat‐flux theory, from 1.6 at *C*_r_  =  10 to 1.7 at *C*_r_  =  50. At conditions of *C*_r_ = 40, *Z* = 2 × 10^−3^ K^−1^, and *T*_c,oc_  =  285.4 K, the peak value of *η*_TE_ increases largely as the material's *E*_g_ increases, from 0.9% at *E*_g_  =  0.66 eV to 9.5% at *E*_g_  =  1.81 eV, while the *s*_opt_ slightly increases from 1.6 at *E*_g_  =  0.66 eV to 1.8 at *E*_g_  =  1.81 eV. This indicates that the *s*_opt_ is mainly influenced by *ZT* while heat flux also affects the *s*_opt_ in constant heat‐flux theory.

**Figure 6:**
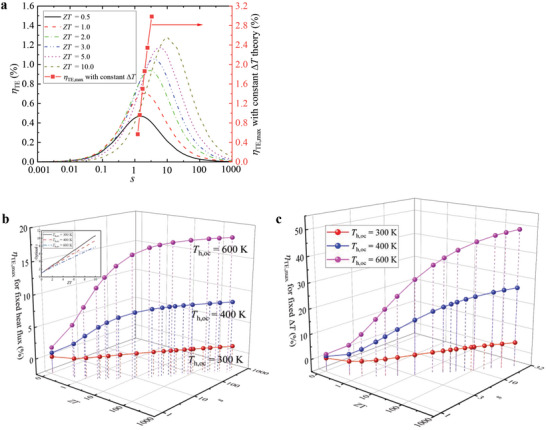
TE efficiencies obtained with constant heat‐flux and temperature‐difference theories. a) TE efficiencies obtained with the two theories at night, *T*_h,oc_ = 300 K. b) Maximum TE efficiency obtained with constant heat‐flux theory. c) Maximum TE efficiency obtained with constant ∆*T* theory. Other condition: *T*_c,oc_  =  283.53 K.

For a given ∆*T*_oc_, the *η*_TE_ obtained with constant heat‐flux and temperature‐difference theories are presented in **Figure** **6**. At night, the temperature on the cold side reaches 283.53 K via the radiative cooler, while the temperature on the hot side keeps the same as the ambient temperature (*T*_a_ = 300 K). In this case, the ambient is employed as the heat source whose heat capacity is very large, and the TEG is viewed as operating under a constant temperature‐difference condition (*T*_h,oc_ = 300 K and *T*_c,oc_ = 283.53 K), and its efficiency is shown in Figure 6a. The peak value of *η*_TE_ obtained with constant heat‐flux theory increases as *ZT* rises, from 0.5% at *ZT*  =  0.5 to 1.3% at *ZT* = 10, and the corresponding *s*_opt_ increases from 1.5 at *ZT* = 1.5 to 10.7 at *ZT* = 10. The *η*_TE,max_ obtained with constant ∆*T* theory increases greatly from 0.6% at *ZT* = 0.5 to 3% at *ZT*  =  10, and the corresponding *s*_opt_ increases from 1.2 at *ZT* = 0.5 to 3.3 at *ZT* = 10 following Equation (10). It is clear that the *η*_TE,max_ obtained with constant ∆*T* theory is higher than that obtained with the constant heat‐flux theory, and the absolute difference between the two *η*_TE,max_ increases as *ZT* increases, from 0.1% at *ZT* = 0.5 to 1.7% at *ZT*  =  10.

The temperature on the cold side remains unchanged at *T*_c,oc_ = 283.53 K, the temperature on the hot side increases to 400 and 600 K (i.e., *T*_h,oc_ = 400 K and *T*_h,oc_ = 600 K), the *η*_TE,max_ obtained with the constant heat‐flux and ∆*T* theories as a function of *ZT* and *s* are presented in Figure 6b,c. In the constant heat‐flux theory, the *s*_opt_ increases as *ZT* increases for a given *T*_h,oc_, and also slightly increases as *T*_h,oc_ increases for a given *ZT*. For example, the *s*_opt_ increases from 1.9 at *ZT* = 1 to 9.5 at *ZT* = 10 with *T*_h,oc_ = 400 K, and decreases slightly from 2 at *T*_h,oc_ = 300 K to 1.8 at *T*_h,oc_ = 600 K with *ZT* = 1. In constant ∆*T* theory, the *s*_opt_ slightly increases with *ZT*, and is not affected by *T*_h,oc_. In constant heat‐flux theory, the *η*_TE,max_ increases significantly as *ZT* increases in the case of *ZT* < 10, while it increases very slightly in the case of *ZT* > 10, so it is more cost‐effective to make *ZT* < 10 in actual design of TEG. In the case of *ZT* < 10, the *s*_opt_ increases approximately linearly with *ZT* in constant heat‐flux theory as shown in Figure 6b, and the fitting formulas between them are as follows

(12) $$\begin{array}{ccc} s_{\mathrm{opt}} & = & 0.97\cdot ZT+1.02,\;\mathrm{at}\;T_{h,\mathrm{oc}}=300\;K\\ s_{\mathrm{opt}} & = & 0.84\cdot ZT+1.09,\;\mathrm{at}\;T_{h,\mathrm{oc}}=400\;K\\ s_{\mathrm{opt}} & = & 0.67\cdot ZT+1.18,\;\mathrm{at}\;T_{h,\mathrm{oc}}=600\;K \end{array}$$

### 2.4 Spectral‐Splitting MJ PV‐TE System

**Figure 7:**
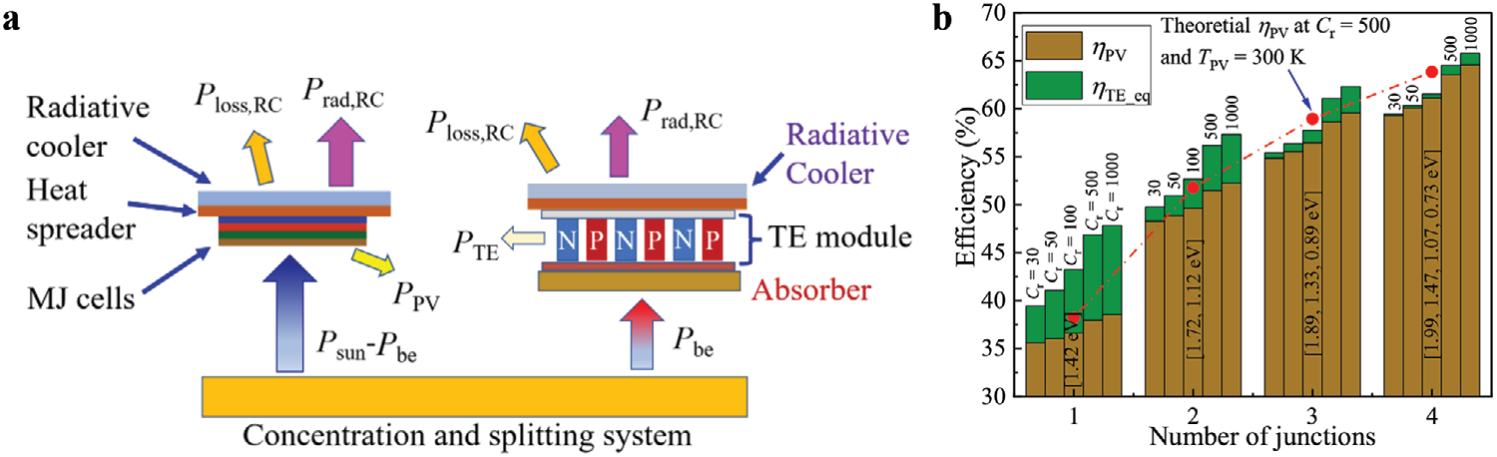
SS MJ PV‐TE‐RC system and its efficiency. a) A concentrated SS MJ PV‐TE‐RC system. b) Efficiencies in these systems with various *C*_r_, at conditions of *R*_c_  =  *C*_r_, *ZT*  =  2, and *ξ*_ab_  =  0.1.

To get as much electricity as possible from the sun, MJ cells are employed to reduce the thermalization heat, namely, the single‐junction PV cell in Figure 3 is replaced by MJ cells (as shown in **Figure** **7**a). The *E*_g_ of MJ cells given in ref. [37] is adopted in this section, which is listed in Figure 7b. In this SS MJ PV‐TE system, the solar spectrum with photon energy below the minimum *E*_g_ is directed to the absorber to be utilized by TEG. The photons with energy above the minimum *E*_g_ are utilized by the cell with matching *E*_g_, and the thermalization heat is dissipated through the radiative cooler placed above the PV panel.

Five concentration ratios (*C*_r_  =  30, 50, 100, 500, and 1000) are selected to investigate the efficiency of the SS MJ cells system, where *C*_r_  =  100 is taken as a typical concentration ratio in a current parabolic trough or parabolic dish, *C*_r_  =  1000 is taken as a typical concentration ratio of current solar towers,^[^ ^38^ ^]^ and *C*_r_  =  500 is taken as typical moderate concentration ratio. Although *C*_r_  =  46 000 is generally taken as the maximum concentration ratio achievable on earth,^[^ ^39^ ^]^ *C*_r_ = 1000 is taken as the maximum concentration ratio in this study, considering the temperature that materials can withstand, the uniformity of temperature in heat spreader of radiative cooler, cost, covered area, etc. The *η*_TE_eq_ decreases as the number of junctions increases, while increases as *C*_r_ increases. For example, at *C*_r_  =  50, *η*_TE_eq_ is 5% for one junction, 2.1% for two junctions, 0.8% for three junctions, and 0.2% for four junctions. In the system with two‐junction cells, *η*_TE_eq_ increases from 1.5% at *C*_r_  =  30 to 5.1% at *C*_r_  =  1000. In the system with four‐junction cells, *η*_TE_eq_ is about 1.2% at *C*_r_ = 1000, so it is less significant to discuss the MJ PV‐TE system when the number of junctions is higher than 4. *η*_PV_ and *η*_TE_eq_ increase as *C*_r_ and the number of junctions increase, while the increasing margin recedes as the number of junctions increases. The theoretical efficiency of PV panel at *C*_r_  =  500 and *T*_PV_  =  300 K is employed as a reference efficiency. In the single‐junction cell system, *η*_PV+TE_ at *C*_r_  =  30 is 39.4% that is higher than the theoretical *η*_PV_ at *C*_r_ = 500 and *T*_PV_  =  300 K (38.2%); and *η*_PV+TE_ at *C*_r_  =  500 is 55.6% that is an absolute increment of more than 17 percentage points relative to the reference efficiency. In the two‐junction cells, *η*_PV+TE_ at *C*_r_ = 100 is 52.7% that is higher than the theoretical *η*_PV_ at *C*_r_ = 500 and *T*_PV_ = 300 K (51.8%), and *η*_PV+TE_ at *C*_r_ = 500 is 56.2% that is an absolute increment of more than 4.4 percentage points relative to the reference efficiency. In the three‐junction cells, *η*_PV+TE_ at *C*_r_ = 500 is 61.1% which is an absolute increment of more than 2.2 percentage points relative to the reference efficiency (58.9%). In the four‐junction cells, *η*_PV+TE_ at *C*_r_ = 500 is 64.5% which is an absolute increment of more than 0.7 percentage points relative to the reference efficiency (63.8%). This indicates that the SS MJ PV‐TE‐RC system is more suitable for the system with a small number of junctions (≤ 3), this is in line with the actual situation, that is the greater number of junctions leads to soaring cost and manufacturing difficulty. At *C*_r_ = 1000, *η*_PV+TE_ increases from 48% to 66% as the number of junctions rises from 1 to 4, and the *η*_PV+TE_ in the hybrid system including four‐junction cells is over 65%, which outperforms most current photoelectric and thermal power systems.

**Figure 8:**
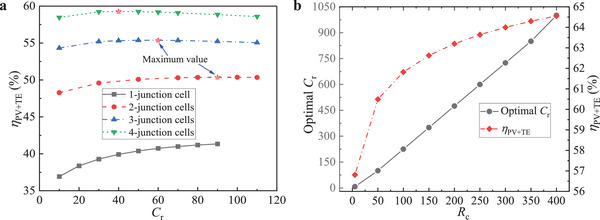
*η*_PV+TE_ in SS MJ PV‐TE‐RC systems. a) *η*_PV+TE_ in the systems as a function of *C*_r_ at *R*_c_  =  25. b) The optimal *C*_r_ that maximizes *η*_PV+TE_ and its corresponding *η*_PV+TE_ as a function of *R*_c_ in the system including four‐junction cells. Other conditions: *ZT* = 2 and *ξ*_ab_  =  0.1.

The *η*_PV+TE_ as a function of *C*_r_ and *R*_c_ is demonstrated in **Figure** **8**. At *R*_c_ = 25 and *ξ*_ab_ = 0.1, as *C*_r_ increases from 10 to 90, *η*_PV+TE_ in the system with one‐junction cell monotonously increases from 36.9% to 41.2% as shown in Figure 8a, while *η*_PV+TE_ in the systems with MJ cells increases first and then decreases. *η*_PV+TE_ in the system with two‐junction cells reaches a peak of 50.4% at *C*_r_  =  90, *η*_PV+TE_ in the system with three‐junction cells reaches a peak of 55.4% at *C*_r_ = 60, *η*_PV+TE_ in the system with four‐junction cells reaches a peak of 59.3% at *C*_r_  =  40. A larger number of junctions in the system means a smaller optimal *C*_r_ that corresponds to the maximum *η*_PV+TE_ for a given *R*_c_. Since the proportion of *η*_PV_ in the *η*_PV+TE_ rises as the number of junctions increases, the changing trend of *η*_PV+TE_ is more dependent on *η*_PV_. In the four‐junction hybrid system, the optimal *C*_r_ at which *η*_PV+TE_ reaches the maximum and its corresponding *η*_PV+TE_ as a function of *R*_c_ are demonstrated in Figure 8b. The optimal *C*_r_ increases nearly linearly as *R*_c_ rises, the ratio of the optimal *C*_r_ to *R*_c_ increases slightly as *R*_c_ increases and the average ratio is 2.3 in the range of parameters selected in this study. *η*_PV+TE_ increases as *R*_c_ rises while the increasing slope declines. Of note is that the *η*_PV+TE_ when optimal *C*_r_ is 1000 is less than *η*_PV+TE_ at *C*_r_  =  1000 in Figure 7b, since *R*_c_ is much higher in the latter (*R*_c_ = 1000) than in the former (*R*_c_ = 400). This indicates that *R*_c_ can be far less than *C*_r_ in practice, but increasing *R*_c_ is one of the most effective ways to improve efficiency.

## 3 Discussion and Conclusions

The bandgap energy (*E*_g_) generally decreases with increasing *T*_PV_, especially at high temperature of *T*_PV_ > 200 °C, which will affect *η*_PV_. If the temperature difference between the ambient and PV cell is not beyond 100 °C, the *η*_PV_ extrapolated based on the *E*_g_ at *T*_a_ is very accurate (almost exactly identical with the *η*_PV_ using *E*_g_ as a function of *T*_PV_) for most PV materials as demonstrated in ref. [40]. The *T*_PV_ is limited to no more than 373 K and *T*_a_ is fixed at 300 K in this study, so it is reasonable to assume that *E*_g_ remains its value at *T*_a_ without variation with *T*_PV_ in this study for the convenience of calculation, and the results are accurate and reliable.

The PV material's *E*_g_ has an important influence on *η*_PV+TE_ as shown in Figure 4a, the *E*_g_ in the systems with MJ cells in Figure 7 is from ref. [37] without considering the addition of TEG. Therefore, *η*_PV+TE_ in SS MJ PV‐TE‐RC system can be further improved by optimizing the combination of *E*_g_ in MJ cells, considering the properties of PV cells and TEG. Heat flux and temperature difference affect each other in practice, there is no definite constant temperature‐difference or heat‐flux condition, just which changes faster. As far as the characteristics of solar energy itself are concerned, it is more reasonable to regard it as a constant heat‐flux condition.

The ∆*T* across TEG in a closed circuit will be lower than that in an open circuit due to the Peltier heat, and the maximum gap occurs when the ratio *s* is 0 (in a short circuit). There exists an optimal *s* in which *η*_TE_ reaches the maximum for a given *ZT*, the optimal *s* increases almost linearly as *ZT* increases, and also increases slightly as *C*_r_ and PV material's *E*_g_ increase.

In the PV‐TE‐RC system with a single‐junction cell in Figure 1d, a temperature lower than *T*_a_ is achieved via a radiative cooler, so the *η*_PV+TE_ (33.8%) is higher than that in the conventional PV‐TE system in Figure 1c (33.1%). Moreover, a *η*_TE_eq_ of 0.4% can be achieved at night. At *R*_c_ = 20, the *η*_tot_ increases and then decreases as *C*_r_ rises, reaches a peak (34.3%) at *C*_r_ = 3, since the increasing *T*_PV_ is inducive to TEG but deteriorates *η*_PV_. The increasing *R*_c_ is beneficial to PV panel and TEG at the same time.

SS PV‐TE‐RC system with a single‐junction cell is more suitable for a high *C*_r_, and the PV material's *E*_g_ has a crucial influence on the TEG. Generally, *η*_TE_ increases as *E*_g_ and *C*_r_ increase, while the system with PV material GaAs (*E*_g_ = 1.42 eV) has the highest *η*_tot_ among the four PV materials (Ge, Si, GaAs, GaInP). For a given *R*_c_, there exists an optimal *C*_r_ at which *η*_PV_ reaches the maximum, while the optimal *C*_r_ that maximizes *η*_PV+TE_ depends on the ratio of *η*_TE_ to *η*_PV_, and a smaller ratio indicates a smaller optimal *C*_r_.

In the SS PV‐TE‐RC system with MJ cells, there exists an optimal *C*_r_ at which *η*_PV+TE_ reaches the maximum for a given *R*_c_ when the number of junctions is not less than 2. The optimal *C*_r_ decreases as the number of junctions increases, and the ratio of optimal *C*_r_ to *R*_c_ increases slightly as *R*_c_ grows. Generally, *η*_PV_ and *η*_PV+TE_ increase as the number of junctions increases while *η*_TE_ decreases quickly. At *C*_r_ = 100, *η*_PV+TE_ in the system with two‐junction cells (52.7%) is higher than the corresponding theoretical *η*_PV_ at *C*_r_ = 500 and *T*_PV_  =  300 K (51.8%). At *C*_r_ = 500, *η*_PV+TE_ in the system with three‐junction and four‐junction cells (61.1% and 64.5%) are higher than the corresponding theoretical *η*_PV_ at *T*_PV_ = 300 K (58.9% and 63.8%), while the gap decreases as the number of junctions increases. *η*_PV+TE_ in a single‐junction cell system at *C*_r_ = 30 (39.4%) is higher than the theoretical *η*_PV_ at *C*_r_ = 500 and *T*_PV_ = 300 K (38.2%), demonstrating the huge advantages of the SS PV‐TE‐RC system, especially when the number of junctions is small. *η*_PV+TE_ is over 65% at *C*_r_ = 1000 in the system with four‐junction cells which is superior to most current photovoltaic and thermal power systems.

## 4 Experimental Section

### Hybrid PV‐TE Model

The present model is based on the following assumptions: 1) the quantum efficiency of the solar cells is 1 and the solar cells are ideal; 2) the losses of optical devices including concentrator and SS filter are negligible; 3) the diurnal ambient temperature (*T*_a_) is fixed at 300 K, and the sunlight is AM1.5 spectral irradiation (as shown in Figure 1a); 4) each photon whose energy exceeds *E*_g_ excites one electron–hole pair, and the excess energy above *E*_g_ dissipates as waste heat in solar cells; 5) the thermal resistances between solar cells and TE module, and between TE module and RC are negligible; 6) the transverse heat loss between the TE module and the ambient is negligible; 7) the solar cells remain the same temperature with the ambient at night.

The photons emitted by a blackbody with temperature *T* according to Planck's law read^[^ ^41^ ^]^

(13) $$\phi_{\mathrm{bb}}\left(E,T\right)=\frac{2\pi }{h^{3}c^{2}}\frac{E^{2}}{\exp\left[E/\left(k_{b}T\right)\right]-1}$$

where *h* is the Plank's constant, *k*_b_ is the Boltzmann constant, *c* is the speed of light, and the subscript “bb” represents blackbody. The spectral energy flux of the black body had the expression

(14) $$I_{\mathrm{bb}}\left(E,T\right)=E\phi_{\mathrm{bb}}\left(E,T\right)$$

where *E* is the photon energy. According to the above assumptions, the thermalization heat of a PV cell read^[^ ^42^ ^]^

(15) $$P_{\mathrm{th}}=\int_{E_{g}}^{\infty }C_{r}A_{\mathrm{PV}}\left(E-E_{g}\right)\phi_{\mathrm{AM}1.5}dE$$

The spectral energy below *E*_g_ that cannot be utilized by PV cell could be written as^[^ ^42^ ^]^

(16) $$P_{\mathrm{be}}=\int_{0}^{E_{g}}C_{r}A_{\mathrm{PV}}E\phi_{\mathrm{AM}1.5}dE$$

Thus, the thermal balance of a PV cell could be written as

(17) $$\begin{array}{ccc} P_{\mathrm{th}}+P_{\mathrm{be}} & = & \varepsilon_{\mathrm{PV}}\sigma \left(2A_{\mathrm{PV}}-N_{\mathrm{TE}}A_{\mathrm{TE}}\right)\left(T_{\mathrm{PV}}^{4}-T_{a}^{4}\right)\\ & & +\;h_{c}\left(2A_{\mathrm{PV}}-N_{\mathrm{TE}}A_{\mathrm{TE}}\right)\left(T_{\mathrm{PV}}-T_{a}\right)+Q_{\mathrm{TE}} \end{array}$$

where *ε* is the emissivity, *σ* is the Stefan–Boltzmann constant, *Q*_TE_ is the heat transferred from PV cell to TE module. The heat transfer coefficient *h*_c_ is mainly affected by wind speed, the relevant expressions of *h*_c_ as a function of wind speed are summarized in refs. [1, 43]. It is generally recommended *h*_c_  =  5 W m^−2^ K^−1^ in light breeze and *h*_c_  =  40 W m^−2^ K^−1^ at strong breeze, *h*_c_ could be reduced by windshield or vacuum in practice. The default *h*_c_ was 5 W m^−2^ K^−1^ for the convenience of calculation in this study, unless otherwise specified. The heat transferred from PV cell to TE module read^[^ ^44^ ^]^

(18) $$Q_{\mathrm{TE}}=\frac{k_{\mathrm{TE}}N_{\mathrm{TE}}A_{\mathrm{TE}}\left[1+Z\left(3T_{\mathrm{PV}}+T_{c}\right)/8\right]\left(T_{\mathrm{PV}}-T_{c}\right)}{L_{\mathrm{TE}}}$$

where *k*_TE_ is the thermal conductivity of TE material (*k*_TE_ = 1.5 W m^−1^ K^−1^).

The current of a single‐junction solar cell under solar irradiance could be written as^[^ ^45^ ^]^

(19) $$J_{\mathrm{PV}}=J_{L}-J_{0}\left[\exp\left(\frac{qV_{\mathrm{PV}}}{nk_{b}T_{\mathrm{PV}}}\right)-1\right]$$

where *J*_L_ is the photogenerated current, *J*_0_ is the reverse saturation current, *n* is the ideal factor with *n*  =  1 for ideal p‐n junctions, *q* is the elementary charge, *V* is the voltage. The open‐circuit (oc) voltage could be deduced from Equation (19) with *J*_PV_  =  0

(20) $$V_{\mathrm{oc}}=\frac{nk_{b}T_{\mathrm{PV}}}{q}\ln\left(\frac{J_{L}}{J_{0}}+1\right)$$

**Figure 9:**
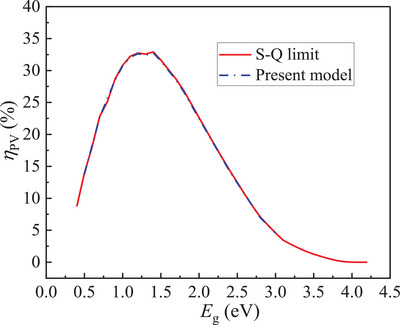
The *η*_PV_ in the present model and the S‐Q limit as a function of *E*_g_.

The ideal PV power could be written as

(21) $$P_{\mathrm{PV}}=V_{\mathrm{oc}}\cdot J_{\mathrm{sc}}\cdot \mathrm{FF}$$

where *J*_sc_ is the short circuit current of PV cell and FF is the fill factor. The PV efficiency obtained in the present model had a good agreement with the well‐known S‐Q limit as shown in **Figure** **9**, validating the reliability of the present model.

### Thermoelectric Efficiency under Constant Heat‐Flux Conditions

For a TE module working at temperature difference ∆*T*, the electrical current read

(22) $$I=\frac{\alpha \cdot \Delta T}{R_{L}+R_{i}}$$

The voltage across the module read

(23) $$V=\frac{N_{\mathrm{TE}}\alpha \cdot \Delta T\cdot s}{1+s}$$

The thermoelectrical conversion efficiency could be written as

(24) $$\eta_{\mathrm{TE},\Delta T}=\frac{\left(1-\frac{T_{c}}{T_{h}}\right)s}{\left(1-s\right)-\frac{1}{2}\left(1-\frac{T_{c}}{T_{h}}\right)+\frac{1}{2ZT}{\left(1-s\right)}^{2}\left(1+\frac{T_{c}}{T_{h}}\right)}$$

The maximum conversion efficiency could be obtained by differentiating Equation (24) with respect to the ratio *s* and setting it to zero, yielding Equations (9) and (10) above.

However, Equation (9) would become invalid under constant heat‐flux condition, to address this issue, a new theory was established for TEG operating under constant heat‐flux condition recently.^[^ ^36^ ^]^ The key to the new theory was the relationship between the ∆*T* across TEG under open‐circuit and closed‐circuit conditions, which was written as^[^ ^36^, ^44^ ^]^

(25) $$\Delta T_{\mathrm{oc}}=\Delta T_{\mathrm{cc}}\left(1+ZT_{m}\right)$$

with

(26) $$T_{m}=\frac{\left(1+2s\right)T_{h,cc}+T_{c,cc}}{2{\left(1+s\right)}^{2}}$$

where subscript “oc” and “cc” denote open circuit and closed circuit, and subscript “h” and “c” denote hot and cold. Therefore, the voltage and current under heat‐flux condition in a closed circuit could be written as

(27) $$V_{\mathrm{cc}}=\frac{\alpha \Delta T_{\mathrm{oc}}}{1+ZT_{m}}\frac{R_{L}}{R_{i}+R_{L}}$$

(28) $$I_{\mathrm{cc}}=\frac{\alpha \Delta T_{\mathrm{oc}}/\left(1+ZT_{m}\right)}{R_{i}+R_{L}}$$

Finally, the TE power and TE efficiency could be written as follows^[^ ^36^ ^]^

(29) $$P_{\mathrm{TE}}=\frac{{\left(\alpha \Delta T_{\mathrm{oc}}\right)}^{2}s}{R_{i}{\left(1+ZT_{m}\right)}^{2}{\left(1+s\right)}^{2}}$$

(30) $$\eta_{TE}=\frac{Z\Delta T_{\mathrm{oc}}s}{{\left(1+ZT_{m}\right)}^{2}{\left(1+s\right)}^{2}}$$

**Figure 10:**
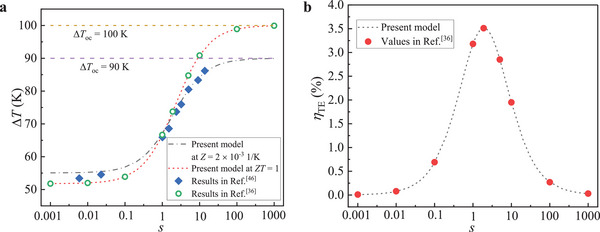
Comparison between the results obtained in the present model and in literatures. a) The temperature differences under a closed‐circuit state obtained in this study against the results at ∆*T*_oc_  =  90 K in ref. [46] and at ∆*T*_oc_ = 100 K in ref. [36]. b) TE efficiency obtained in the present work against the results at ∆*T*_oc_ = 100 K in ref. [36].

The temperature difference and efficiency of TEG obtained in this study against the results in literature are presented in **Figure** **10**. At ∆*T*_oc_ = 100 K, the temperature difference and efficiency obtained in this study had good agreement with the results in ref. [36]. At ∆*T*_oc_ = 90 K, the temperature difference obtained in this study was in good agreement with the results given in ref. [46] between *s* = 1 and *s* = 10, although the results obtained in this study were slightly higher in the range of *s* < 0.1. Of note was that the results discussed in this study were mainly concentrated between *s* = 1 and *s* = 10, therefore, the calculation results about TEG were reliable and acceptable in this study.

### Radiative Cooling

The thermal energy emitted by a material with an emissivity of *ε*_RC_(*λ*,*θ*) could be written as

(31) $$\begin{array}{c} \begin{array}{ccc} P_{\mathrm{rad},\mathrm{RC}}\left(T_{\mathrm{RC}}\right) & = & \int_{\Omega }\cos\theta \int_{0}^{\infty }R_{c}A_{\mathrm{PV}}\varepsilon_{\mathrm{RC}}\left(\lambda ,\theta \right)I_{\mathrm{bb}}\left(\lambda ,T_{\mathrm{RC}}\right)d\lambda d\Omega \\ & = & 2\pi \int_{0}^{\pi /2}\int_{0}^{\infty }R_{c}A_{\mathrm{PV}}\varepsilon_{\mathrm{RC}}\left(\lambda ,\theta \right)I_{\mathrm{bb}}\left(\lambda ,T_{\mathrm{RC}}\right)\sin\theta \cos\theta d\lambda d\theta \end{array} \end{array}$$

where *θ* denotes the angle between the direction of the solid angle and the normal direction of the surface, *Ω* is the solid angle.

The solar power that was absorbed by the radiative cooler read

(32) $$P_{\mathrm{sun},\mathrm{RC}}=\int_{0}^{\infty }I_{\mathrm{AM}1.5}\left(\lambda \right)R_{c}A_{\mathrm{PV}}\varepsilon_{\mathrm{RC}}\left(\lambda ,\theta_{\mathrm{sun}}=0\right)d\lambda$$

where *θ*_sun_ denotes the direction of incident sunlight. The *θ*_sun_ was assumed to be 0 so as to maximize solar spectral irradiance.

The power absorbed by RC from the atmosphere via radiation could be written as

(33) $$\begin{array}{ccc} P_{\mathrm{atm},\mathrm{RC}}\left(T_{a}\right) & = & 2\pi \int_{0}^{\pi /2}\int_{0}^{\infty }I_{\mathrm{bb}}\left(\lambda ,T_{a}\right)R_{c}A_{\mathrm{PV}}\varepsilon_{\mathrm{atm}}\left(\lambda ,\theta \right)\varepsilon_{\mathrm{RC}}\left(\lambda ,\theta \right)\\ & & \sin\theta \cos\theta d\lambda d\theta \end{array}$$

where *ε*_atm_ is the atmospheric emissivity, which was given by^[^ ^47^ ^]^

(34) $$\varepsilon_{\mathrm{atm}}\left(\lambda ,\theta \right)=1-\tau_{\mathrm{atm}}{\left(\lambda \right)}^{\cos\theta }$$

where *τ*_atm_ is the atmospheric transmittance in the zenith direction.

Lastly, the cooling power of the radiative cooler was written as

(35) $$\begin{array}{ccc} P_{\mathrm{cool}}\left(T_{\mathrm{RC}}\right) & = & P_{\mathrm{rad},\mathrm{RC}}\left(T_{\mathrm{RC}}\right)-P_{\mathrm{sun},\mathrm{RC}}-P_{\mathrm{atm},\mathrm{RC}}\left(T_{a}\right)-Q_{\mathrm{in}}\\ & & -\;h_{c}R_{c}A_{\mathrm{PV}}\left(T_{a}-T_{\mathrm{RC}}\right) \end{array}$$

where *Q*_in_ represents the heat entering the radiative cooler from PV panel or TEG, and the last term on the right of Equation (35) denotes the heat entering from the ambient.

## Conflict of Interest

The authors declare no conflict of interest.

## Data Availability

The data that support the findings of this study are available from the corresponding author upon reasonable request.
